# Small bowel varices secondary to chronic superior mesenteric vein thrombosis in a patient with heterozygous Factor V Leiden mutation: a case report

**DOI:** 10.1186/s13256-015-0705-6

**Published:** 2015-10-01

**Authors:** Maria C. Garcia, Golo Ahlenstiel, Hema Mahajan, David van der Poorten

**Affiliations:** 1 Sydney Medical School, Westmead Clinical School, University of Sydney, Sydney, NSW Australia; 2 Department of Gastroenterology and Hepatology, University of Sydney at Westmead Hospital, Westmead, NSW Australia; 3 Department of Anatomical Pathology, Westmead Hospital, Westmead, NSW Australia

**Keywords:** Ectopic varices, Factor V Leiden, Superior mesenteric vein thrombosis

## Abstract

**Introduction:**

Bleeding ectopic small bowel varices pose a clinical dilemma for the physician, given their diagnostic obscurity and the lack of evidence-based medicine to guide therapy. They often occur in the context of portal hypertension, secondary to either liver disease or extrahepatic causes. Rarely is their presence associated with chronic superior mesenteric vein thrombosis and hereditary coagulopathies.

**Case presentation:**

A 74-year-old white woman, with a heterozygous Factor V Leiden mutation and no underlying liver disease or portal hypertension, presented over the course of 13 months for recurrent episodes of melena and per rectal bleeding. An initial endoscopy showed a clean-based chronic gastric ulcer, while colonoscopies showed multiple, non-bleeding angioectasias which were treated with argon plasma coagulation. Subsequent video capsule endoscopy and double balloon enteroscopy revealed red wale marks overlying engorged submucosal veins in her distal ileum, consistent with ectopic varices. A chronic superior mesenteric vein thrombus, found via computed tomography venogram, was the cause of the ileal varices. She underwent curative surgical resection of the affected bowel, with no re-bleeding episodes 17 months post-surgery, despite needing lifelong anticoagulation for recurrent venous thromboembolisms.

**Conclusions:**

Clinicians should consider ectopic varices in patients who present with obscure gastrointestinal bleeding, even in the absence of portal hypertension or liver disease. In those with a known thrombophilia, patients should be screened for splanchnic thrombosis, which may precipitate ectopic varices.

## Introduction

Ectopic varices are dilated portosystemic collateral vessels occurring in mucosa outside the gastroesophageal region. Although they account for less than 5% of variceal bleeds, their diagnostic obscurity and high risk of rupture portends considerable mortality. Most commonly, ectopic varices are caused by portal hypertension in the context of pre-existing liver disease (such as cirrhosis or hepatocellular carcinoma); albeit, other etiologies independent from portal hypertension do exist [1].

Small bowel varices secondary to mesenteric vein thrombosis are an exceptionally rare phenomenon, with only 15 documented cases in the English literature. Rarer still, is the link between inherited coagulation disorders and the development of small bowel varices [2]. Here we present the first case report of ileal varices caused by chronic superior mesenteric vein (SMV) thrombosis secondary to a heterozygous Factor V Leiden mutation.

## Case presentation

A 74-year-old white woman was admitted to hospital with a 3-month history of progressive dyspnea and lethargy. Imaging studies showed the presence of multiple bilateral pulmonary emboli in the context of a right lower limb deep vein thrombosis. Incidentally, baseline laboratory testing revealed severe iron deficiency anemia with hemoglobin (Hb) of 55g/L and ferritin of 10μg/L. She denied any prior history of melena or per rectal bleeding, but admitted a 2-year history of bloating, upper abdominal pain and chronic diarrhea. Besides four prior episodes of deep vein thrombosis in the context of a known heterozygous Factor V Leiden mutation, her past medical history was otherwise unremarkable. Specifically, she had no history of liver disease, cirrhosis or portal hypertension. Her regular medications included an angiotensin II blocker for hypertension, a statin for dyslipidemia, allopurinol for gout and 100mg of aspirin. On physical examination, she was slightly tachycardic (heart rate 105), tachypneic (respiratory rate 26 breaths per minute) and saturating at 96% on room air. Her blood pressure was normotensive at 117/85mmHg, and she was afebrile.

An endoscopy revealed a large, clean-based 1.5cm chronic gastric ulcer on the lesser curvature of her stomach scattered diverticuli were noted within her sigmoid colon during colonoscopy. There was no evidence of *Helicobacter pylori* on biopsy or urease testing and no bleeding was identified at either procedure. She was discharged on high-dose pantoprazole, and warfarin was recommenced for venous thromboembolism (VTE) prophylaxis. Her international normalized ratio (INR) was monitored and maintained between 2 and 3 (with good patient compliance).

**Fig. 1:**
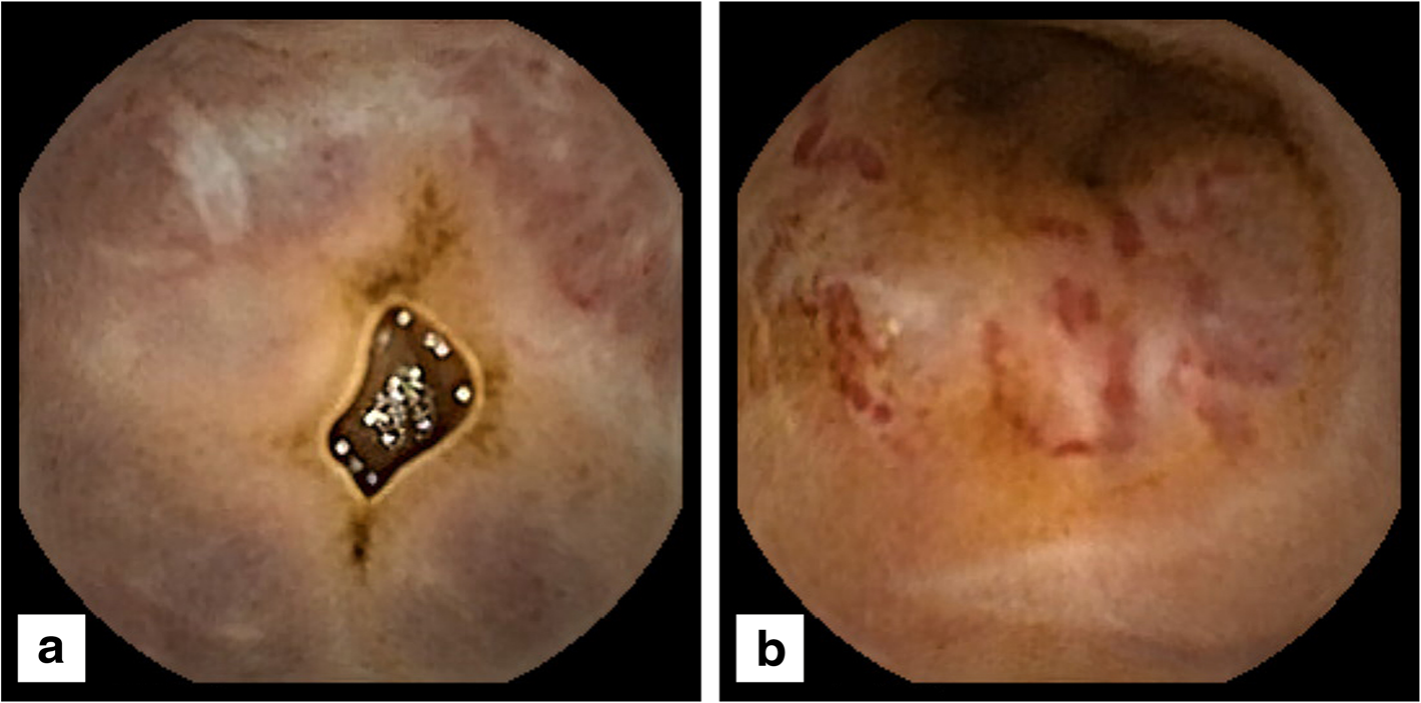
Video capsule endoscopy showing (**a**) prominent submucosal vessels in the distal ileum and (**b**) red wale markings in the distal ileum

Despite repeat gastroscopy showing resolution of her gastric ulcer, over the next 9 months she represented four times for symptomatic melena and per rectal bleeding, which required at least one unit of packed red blood cells for three of her presentations (Hb <70g/L). Repeat colonoscopies showed multiple, non-bleeding angioectasias which were treated with argon plasma coagulation. Subsequently, rapid active bleeding was demonstrated in her distal ileum with a technetium-99m red blood cell scan, which was concordant with subsequent findings of engorged submucosal veins with red wale marks on capsule endoscopy (Figs. 1a and 1b) and double balloon enteroscopy.

**Fig. 2:**
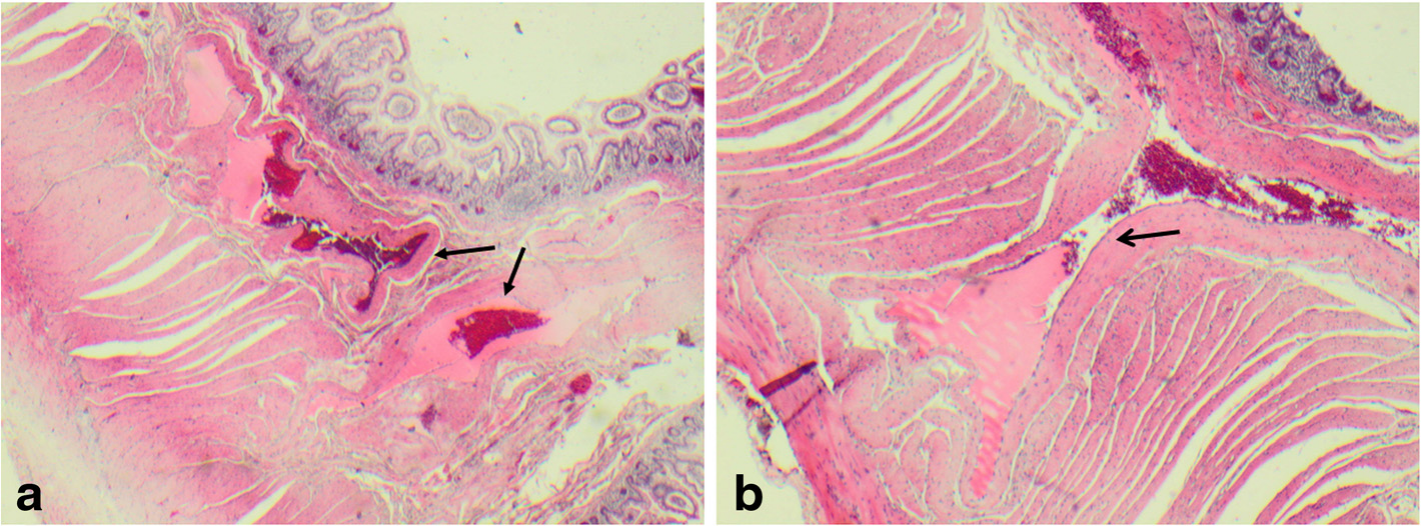
**a** Abnormal tortuous vessels (*arrows*) in submucosa, extending into muscularis propria (hematoxylin-eosin, ×2); and **b** tortuous vessel (*arrow*) extending into muscularis propria (hematoxylin-eosin, ×4)

Further investigation with a computed tomography (CT) venogram showed obscuration of her superior mesenteric vein (SMV) with surrounding collaterals, suggestive of chronic thrombosis. A surgical opinion was sought regarding therapeutic options, and the patient underwent an elective small bowel resection of 15cm of the distal ileum. At operation, marked varices were noted on her bowel wall, as well as thickened mesentery, which was consistent with SMV thrombosis. Histopathological findings were in keeping with a vascular lesion, as tortuous and collapsed venules and veins were present within the submucosa and muscularis propria (Figs. 2a and 2b).

Her protracted admissions were compounded by the necessity of anticoagulation therapy, given her history of recurrent VTEs. Seventeen months post-surgery, however, she has not experienced any further episodes of gastrointestinal (GI) bleeding and her Hb and iron studies have normalized, despite needing lifelong anticoagulation with warfarin.

## Discussion

Ectopic varices are rare causes of GI bleeding, comprising 1 to 5% of variceal bleeds and 1 to 2% of obscure GI bleeding. Although two-thirds of ectopic varices are located in the small intestine, only 7 to 12% of bleeding arises from the jejunum or ileum [3, 4]. As such, bleeding ectopic varices distal to the ligament of Treitz can pose a diagnostic dilemma for the clinician.

The majority of patients with ectopic varices have portal hypertension in the context of cirrhosis or portal vein thrombosis [5]. It is generally uncommon for ectopic varices to be caused by localized portal hypertension in the absence of liver disease. Other less common etiologies include: familial varices, adhesions from prior surgery, pancreatic tumors and mesenteric vein thrombosis [6]. There are only 15 documented cases in the literature reporting small bowel varices secondary to SMV thrombosis, of which three are caused by inherited coagulopathies [2, 7, 8]. Although there is a clear link between inherited thrombophilia and small bowel varices, our case report is the first to describe SMV thrombosis in the context of a ‘heterozygous’ Factor V Leiden mutation.

The Factor V Leiden mutation is the most prevalent cause of inherited thrombophilia in whites and occurs in 5 to 12% of the general population. The substitution of arginine for glutamine at position 506 alters the activated protein C cleavage site on Factor V and results in a 10- to 20-fold slower inactivation of Factor Va. Heterozygous carriers have a threefold to sevenfold risk of VTE compared to non-carriers; heterozygous carriers have a 10% chance of developing a VTE within their lifetime, and have a 1.41 odds ratio for developing recurrent VTEs. Typically, mesenteric vein thrombosis in the context of heterozygous Factor V Leiden mutation presents as an acute abdomen, rather than an indolent course [9, 10]. Although unusual, it should be noted that heterozygous carriers are at risk of developing chronic SMV thrombosis and, as such, ectopic varices.

The final diagnosis and etiology in our patient was confirmed by video capsule endoscopy, double balloon enteroscopy and CT venogram. Capsule endoscopy is a relatively non-invasive test, capable of capturing two to six frames per second of small bowel mucosa. Images are not visualized in real-time; however, the pitfalls of this are easily mitigated by double balloon enteroscopy. Diagnostic yields in obscure GI bleeding for capsule endoscopy and double balloon enteroscopy are 62 and 56%, respectively, and when used in combination result in significantly higher diagnostic yields of 75% [11]. Chronic SMV thrombosis can be differentiated from an acute occlusion by the presence of extensive venous collaterals and/or the inability to visualize the SMV on CT [12].

Due to the infrequency of bleeding ectopic varices, optimal treatment is currently dictated by case reports and small retrospective case series. Several interventional modalities exist for treating ectopic varices; however, when used as monotherapy they are associated with re-bleeding. Moderate re-bleeding rates of 16 to 37% have been reported for transjugular intrahepatic portosystemic shunts (TIPS) [13] and whilst balloon-occluded retrograde transvenous obliteration (BRTO) seems promising (with re-bleeding rates of 5% at 24 months) [14], there generally is a paucity of long-term follow up beyond a year in the literature [15]. Both TIPS and BRTO are unique interventions associated with limitations and risks [16]; notably, they may not provide adequate variceal decompression, and there is the potential that varices at other sites not treated with sclerosant may bleed [17].

Endoscopic techniques and interventional radiology are recommended first and second line therapy (respectively) for bleeding ectopic varices, with a view for surgery if bleeding is unamenable to treatment and the patient has favorable liver function [6]. Given that BRTO is not an available treatment modality at our institution, and the localized nature of the small bowel varices, surgery seemed a potential recourse for our patient. Variceal bleeding was successfully resolved via resection of the affected bowel, with no complications or re-bleeding 17 months post-surgery.

## Conclusions

In conclusion, ectopic varices are an important differential for patients with obscure GI bleeding, even when there is no history of liver disease or portal hypertension. Although uncommon, Factor V Leiden heterozygous carriers are at risk of developing chronic SMV thrombosis and consequential ectopic varices. In instances where TIPS or BRTO are not feasible, surgery should be considered a viable option for treating localized ectopic varices in patients with no history of liver disease or other comorbidities.

## Consent

Written informed consent was obtained from the patient for publication of this case report and accompanying images. A copy of the written consent is available for review by the Editor-in-Chief of this journal.
